# Mesenchymal stem cell therapy and acute graft-versus-host disease: a review

**DOI:** 10.1007/s13577-014-0095-x

**Published:** 2014-06-06

**Authors:** Bruna Amorin, Ana Paula Alegretti, Vanessa Valim, Annelise Pezzi, Alvaro Macedo Laureano, Maria Aparecida Lima da Silva, Andréa Wieck, Lucia Silla

**Affiliations:** 1 Cell Therapy Center, Experimental Research Center, Hospital de Clínicas de Porto Alegre, Porto Alegre, Rio Grande do Sul Brazil; 2 Graduate Program in Medicine: Medical Sciences, Universidade Federal do Rio Grande do Sul (UFRGS), Porto Alegre, Brazil; 3 Department of Hematology, Hospital de Clínicas de Porto Alegre, Porto Alegre, Rio Grande do Sul Brazil; 4 Hospital de Clinicas de Porto Alegre, Ramiro Barcellos, 2350, Bairro Santa Cecília, Porto Alegre, CEP 90035-903 Brazil

**Keywords:** Mesenchymal stem cell, Graft-versus-host disease, Immunomodulation, Cell therapy, Inflammatory

## Abstract

Mesenchymal stem cells (MSCs) are being widely studied as potential cell therapy agents due to their immunomodulatory properties, which have been established by in vitro studies and in several clinical trials. Within this context, mesenchymal stem cell therapy appears to hold substantial promise, particularly in the treatment of conditions involving autoimmune and inflammatory components. Nevertheless, many research findings are still contradictory, mostly due to difficulties in characterization of the effects of MSCs in vivo. The purpose of this review is to report the mechanisms underlying mesenchymal stem cell therapy for acute graft-versus-host disease, particularly with respect to immunomodulation, migration, and homing, as well as report clinical applications described in the literature.

## Stem cells

By definition, stem cells are undifferentiated cells with the capacity to undergo self-renewal by means of asymmetric mitotic division [1]. The main characteristics of stem cells that make them extremely appealing for cell therapy are their aforementioned capacity for self-renewal, i.e., their ability to multiply while remaining undifferentiated, thus enabling constant, active replacement of cell populations in tissues, and their potential ability to differentiate into a variety of distinct cell types [2].

Stem cells can be broadly divided into two groups by site of origin: embryonic stem cells (ESCs), which are derived from the inner cell mass of a blastocyst, and adult stem cells (ASCs), which are obtained from umbilical cord blood, bone marrow, or peripheral blood, and present in specific tissues and organs throughout the adult body [3–6].

Totipotent stem cells are the only cell type capable of originating an entire organism, as they are able to generate all cell and tissue types, including both embryonic and extraembryonic tissues (such as the placenta) [7]. Pluripotent stem cells, in turn, are able to differentiate into cells from any of the three primary germ layers (ectoderm, mesoderm, and endoderm, primordial tissues formed in the early stages of embryonic development that will later originate all other tissues in the body). Unlike totipotent cells, pluripotent cells cannot grow an entire organism, as they are incapable of generating extraembryonic tissues [8].

ASCs remain in a quiescent or low-proliferation state, mostly in phases G0 and G1 of the cell cycle, and are located in specific regions that ensure their development and the maintenance of their attributes, particularly their capacity for self-renewal [9]. These regions are known as stem cell niches, and their main sites include the bone marrow [10], heart [11], kidneys, skin, liver, pancreas, ovaries, umbilical cord, placenta, and amniotic fluid [12].

Bone marrow hematopoietic stem cells (HSCs) were the first ASCs to be studied and, consequently, are the best characterized. These cells are capable of differentiation into the myeloid and lymphoid components of blood, and their transplantation has long been used to great effect in the treatment of bone marrow failure and cancer [13].

Another type of ASC present in the bone marrow, but with distinct properties from those of HSCs, was later isolated: mesenchymal stem cells (MSCs), also known as stromal stem cells [14]. As reported at the time of their discovery by Friedenstein in the 1970s, MSCs are highly plastic adherent and are similar to fibroblasts. As multipotent stem cells, MSCs can differentiate into cells derived from the mesoderm germ layer, namely chondroblasts, adipocytes, and osteocytes [15]. In vitro, culture-expanded MSCs express membrane antigens that can be immunophenotyped by flow cytometry. The most widely accepted antigen expression pattern is CD29, CD105, CD73, and CD90 positivity in ≥95 % of cells and minimal expression of CD45, CD34, CD3, CD14, CD19, or HLA-DR, which should be positive in less than 2 % of cells [16, 17].

As they inhibit the proliferation and cytotoxic action of immune cells, MSCs have been employed in the clinical treatment of several diseases, including graft-versus-host disease (GVHD) in its acute form [18]. The purpose of this review is to report the mechanisms underlying MSC therapy for acute GVHD (aGVHD) as they relate to immunomodulation, migration, and homing and to describe clinical applications for MSC therapy that have been previously reported in the literature.

## Bone marrow transplantation and acute graft-versus-host disease

Allogeneic hematopoietic stem cell transplantation (HSCT) is a potentially curative treatment option and treatment of choice for several malignant and nonmalignant conditions, particularly those affecting the hematopoietic system. However, HSCT is associated with high morbidity and mortality rates, and GVHD is the foremost serious complication of this treatment modality [19, 20].

Chronic GVHD (cGVHD) is related to late mortality and is the leading cause of morbidity in long-term survivors of allogeneic HSCT. Symptoms usually present within the first year and are often preceded by an episode of aGVHD. Its clinical manifestations are similar to those of several autoimmune or immune system disorders, such as scleroderma, primary biliary cirrhosis, immune cytopenias, and chronic immunodeficiency and may be limited to a single organ system or may be generalized. cGVHD can have debilitating consequences, including joint contractures, vision loss, end-stage lung disease, and profound chronic immunosuppression [21].

aGVHD remains a major cause of immediate morbidity and mortality in allogeneic HSCT recipients, even when donor and recipient have a high level of human leukocyte antigen (HLA) compatibility [22]. aGVHD commonly affects the skin, gastrointestinal tract (GI), and liver, and usually presents within 100 days of allogeneic HSCT. The pathophysiology of aGVHD is characterized by three well-established stages. The first, commonly known as “cytokine storm”, is a result of the HSCT conditioning regimen. The second involves cellular activation, and is characterized by activation of donor T cells by recipient cytokines and antigen-presenting cells (APCs). The third is the “effector” stage, in which T cells start to damage the cells of certain host tissues [22].

Current prophylaxis regimens for GVHD usually combine a calcineurin inhibitor (cyclosporin A [CsA] or tacrolimus) and a short course of methotrexate (MTX) [23]. There is no clear consensus on optimal prophylaxis for high-risk patients and recipients of unconventional grafts (inadequate donor, older patients, reduced-intensity conditioning regimen), and several other immunosuppressants have been employed, including sirolimus plus tacrolimus and low-dose (5 mg/m^2^) MTX [24]. The efficacy of mycophenolate mofetil (MMF) plus CsA has been studied, especially in patients who underwent reduced-intensity conditioning (RIC) regimens. MMF may replace MTX as an adjunct to CsA, due to lower rates of mucositis and good overall tolerability [25]. Recipients of mismatched grafts usually require more intensive immunosuppression. Both ex vivo and in vivo methods for T-cell depletion (TCD), the latter including anti-thymocyte globulin and alemtuzumab, have been employed. These methods usually reduce the incidence of aGVHD, but at the expense of an increased incidence of infection (due to delayed reconstitution of the immune system) and relapse (due to blunting of the GVL effect) [26].

## Treatment of acute GVHD

As the etiology of aGVHD involves an allogeneic cytotoxic reaction of donor lymphocytes, the cornerstone of aGVHD treatment is immunosuppression, with the purpose of inducing donor/recipient tolerance without eliminating the graft-versus-leukemia/graft-versus-lymphoma (GVL) effect [27]. aGVHD can be classified in 4 degrees of severity from I to IV, and systemic treatment is warranted in Grade ≥II. Again, corticosteroids are the first-line therapy of choice [28], and a recent study confirmed that early response to corticosteroids is associated with increased odds of survival [22]. Patients who responded to high-dose steroids within 5 days of treatment initiation had a 27 % mortality rate, versus 49 % in patients who required protracted, high-dose steroid therapy (3). However, only 60–70 % of patients with aGVHD respond to standard corticosteroid therapy. Patients with severe or steroid-refractory aGVHD have few therapeutic alternatives, no established treatment protocol, and a two-year survival as low as 10 % [29].

The first-line treatment of choice for aGVHD is methylprednisolone (MP) at a dose of 2 mg/kg/day. In case of treatment failure, several agents may be used as second-line therapy, such as tacrolimus, MMF, sirolimus (if not used for prophylaxis), anti-thymocyte globulin, monoclonal antibodies (anti-IL-2 receptor, anti-TNFα, anti-CD52, anti-CD147, and anti-CD3), and extracorporeal photopheresis (PUVA) [29–31].

Recent studies of MSCs in the treatment of aGVHD have been the subject of substantial attention due to their promising findings [32–35]. However, there is a dearth of phase III comparisons of these agents in the treatment of steroid-refractory aGVHD and also how to choose the right source of MSCs or how to modulate these cells, because these points are still unclear [28, 36].

## MSCs in the treatment of acute GVHD

A promising treatment option for aGVHD consists of infusion of third-party, HLA-unrelated, or related bone marrow donor MSCs. MSCs have been used in the treatment of aGVHD due to their inhibitory effects on the proliferation and cytotoxic activity of immune system cells [37]. The first trial using mesenchymal progenitor cells was conducted in 1995, in which 15 patients have benefited from administration of autologous bone marrow-derived MSC [38].

Le Blanc et al. [39] reported the result of haploidentical MSC infusion in a 9-year-old boy with grade IV aGVHD of the GI tract and liver. The clinical response was striking, and the patient remained well at 1-year follow-up. A subsequent study was reported by Ringdén et al. in 2006. The authors administered MSCs to eight patients with grade III/IV steroid-resistant aGVHD and one patient with cGVHD. There was complete resolution of aGVHD in 6 out of 8 patients, and survival was significantly longer than in the control group (16 patients with Grade II–IV, treatment-resistant GVHD of the GI tract who did not receive MSC infusions); 5 patients remained alive over a follow-up period of 2 months to 3 years post-infusion [40]. Since then, a wide range of clinical trials have been conducted to test safety and feasibility (phase I), obtain proof of efficacy in human subjects (phase II) and compare MSC therapy versus the standard of care (phase III). These studies have generally shown good tolerability, with no adverse effects of MSC therapy, and encouraging partial or complete response rates [32, 40–44]. Among those, a multicenter trial conducted by Le Blanc et al. described 55 patients treated with MSCs in several European countries. All subjects had grade II–IV, steroid-resistant aGVHD. Overall, 52 % of patients responded to MSC infusions, regardless of HLA compatibility, since of the 92 infusions administered, 69 were prepared from the cells of healthy, unrelated, and HLA-mismatched donors [33].

In the 2010 meeting of the American Society of Blood and Marrow Transplantation, Kurtzberg et al. reported on the use of allogeneic MSCs for treatment of severe steroid-refractory GVHD. The investigators obtained a 64 % response rate in 59 children at 28 days after infusion, and this response was found to correlate with 100-day survival, suggesting that MSC therapy has an excellent risk–benefit ratio [45].

Prasad et al. reported on the use of the shelf allogeneic MSCs for the compassionate treatment of severe steroid-refractory GVHD including 12 children with treatment-resistance grade III and IV gut aGVHD. Overall, 7 (58 %) patients had complete response, 2 (17 %) partial response, and 3 (25 %) mixed response. Complete resolution of GI symptoms occurred in 9 (75 %) patients. Five of 12 patients (42 %) were still alive after a median follow-up of 611 days (range 427–1111). No infusional or other identifiable acute toxicity was seen in any patient [46].

Utilizing the same cellular product, Martin et al. presented the results of a randomized, placebo-controlled, multicenter phase III trial of MSC therapy for treatment of steroid-resistant/refractory aGVHD in 244 patients at the 2010 ASMBT meeting. Although the response rate 28 days after MSC infusion was not significantly higher in the treatment group, subgroups analysis showed a significant higher response rate among patients with liver and bowel involvement [47].

Table 1 provides a summary of the many clinical trials of MSC therapy for aGVHD that have been conducted thus far, as well as the response rates observed. Overall, these studies have shown that MSC infusion appears to be a safe treatment option for aGVHD and is not associated with any long-term risk.

**Table 1:** Clinical studies of MSCs for treatment of acute graft-versus-host disease

References	Study type	*N*	MSC source	P/Media	MSC dose/kg	Response
Le Blanc et al. [39]	Case report	1	BM; Related donor	3 weeks/BFS	1.0 × 10 (6)	Complete response. Patient still alive 1 year after infusion
Ringden et al. 2006/ [40]	Phase I	8	BM; HLA-identical, haploidentical, and unrelated	1–4/FBS	1.0 × 10 (6)	5 patients survived 2 months to 3 years after infusion
Fang et al. [124]	Efficacy	6	Adipose tissue; Haploidentical (2) and unrelated donors (4)	5/FBS	1.0 × 10 (6)	aGVHD resolved in 5 patients; of these, 4 remained alive after a median 40-month follow-up
Muller et al. [125]	Safety and feasibility	7 (2 with aGVHD)	BM;HSC donor (5) and third-party related donors (2)	Max 6 weeks/FBS	0.4 × 10 (6) to 3.0 × 10 (6)	2 patients with severe aGVHD did not progress to cGVHD; one experienced complete remission
Le Blanc et al. [33]	Phase II	55	BM;HLA-identical; HLA-haploidentical; third party.	1–4/FBS	1.4 × 10 (6)	Complete response in 63 % of children and 43 % of adults; partial response in 16 % of children and 17 % of adults; 3 relapses; 2-year survival: 53 %
von Bonin [44]	Cohort	13	BM; Third-party HLA-mismatched donor	1–2/PL	Median: 0.9 × 10 (6)	2 patients (15 %) responded and required no further immunosuppressant therapy. 11 patients received immunosuppressants concomitantly with MSCs; after 28 days, 5 of these (45 %) had responded. 4 patients (31 %) remained alive at 257-day follow-up
Kebriaei et al. [41]	Phase II multicenter RCT	31	Osiris therapeutics; unrelated donors	–	2 or 8 × 10 (6) in combination with corticosteroids	Complete response in 77 %, partial response in 16 % of patients
Lucchini et al. [42]	Multicenter	11	BM; unrelated HLA-mismatched donors	–/PL	Median: 1.2 × 10 (6)	Overall response in 71.4 % of patients, CR in 23.8 % of cases. No patients presented GVHD progression after MSC administration, but 4 patients presented GVHD recurrence 2–5 months post-infusion. 2 patients developed limited cGVHD
Arima et al. [50]	Pilot	3	BM; related; HLA-identical (1); unknown	3-week culture/Expanded with donor serum	5.0 × 10 (6)	Complete response in 1 patient, partial response in 2 patients

*CR* complete response, *FBS* fetal bovine serum, *GI* gastrointestinal, *PBSCT* peripheral-blood stem cell transplantation, *PL* platelet lysate, *PR* partial response

Although the aforementioned studies suggest that MSC administration can provide several benefits in patients with grade II–IV, steroid-resistant aGVHD, caution is necessary as there may be a trend toward selective publication of positive trials in this field. Other large randomized controlled trials (RCTs) are ongoing and should better characterize and assess the impact of this treatment modality.

Infused MSC systemic distribution was studied by Von Bahr et al. which examined 108 tissue samples obtained postmortem from 18 patients who had received HLA-mismatched MSCs. There were no signs of ectopic tissue formation or MSC-derived malignancies on gross or histopathological examination. Donor MSC DNA was detected by PCR in some tissues—including lymph node, lung, and bowel—of 8 patients. Detection of donor DNA correlated negatively with time since infusion and time to sample collection, and there was no correlation between MSC engraftment and treatment response [48].

Regarding the optimal dose of MSCs for infusion, a phase II trial sponsored by Osiris Therapeutics assessed infusion of MSCs obtained from HLA-mismatched third-party donors for the treatment of grade II–IV aGVHD. Patients were randomly allocated to receive either low-dose (2 × 10^6^ cells/kg) or high-dose (8 × 10^6^ cells/kg) MSC infusions. The complete response rate at 28-day follow-up was 77 % in 31 evaluable patients. The authors failed to show a dose–response relationship [41].

On the other hand, some investigators have reported less encouraging outcomes with MSC therapy. A recent retrospective cohort study by Forslöw et al. [49] found that administration of MSCs may be a risk factor for pneumonia-related mortality after HSCT. Some authors believe these negative outcomes are primarily attributable to the heterogeneity of patient populations treated with different HSCT regimen, severity of aGVHD, differences in the source of MSCs cells obtained from a single donor or multiple donors (HLA-related or otherwise), and from bone marrow or adipose tissue and to the use of products of animal origin as cell culture media (such as fetal bovine serum, FBS) [44, 50]. Anti-FBS protein antibodies have been detected in some patients who received MSCs expanded in FBS medium [44]. One possible solution is replacement of FBS with platelet-rich human serum, also known as platelet lysate (PL), which contains the nutrients required for expansion of MSCs in culture. In vitro studies have shown that PL is as effective as FBS for MSC expansion [44, 51], and in vivo studied in humans have also demonstrated successful results [44]. Therefore, as a cell expansion medium, PL is safer from a biological standpoint and noninferior in efficacy to FBS.

## MSCs for prophylaxis of acute GVHD

Some clinical trials have sought to determine the potential role of MSCs in aGVHD prophylaxis, on the basis of preclinical trials attempting to reduce the incidence of aGVHD in murine models of allogeneic HLA-mismatched transplantation [52]. The protocols of these trials have usually entailed co-transplantation of HSCs and third-party MSCs or transplantation of both cell types from the same donor. According to Baron et al. and Lazarus et al., this procedure is safe and appears to reduce mortality [34, 53], but these findings should be interpreted with caution due to small sample sizes and to a lack of controlled cohort studies.

Ning et al. raised the hypothesis of an excessive recurrence rate when HLA-identical sibling-matched HSCs were co-transplanted with MSCs in patients with hematological malignancies. Even so, among the 25 patients enrolled in this open-label, randomized clinical trial, the incidence of grade II–IV aGVHD was lower in the MSC group (11.1 %) than in the control group (53.3 %) [54]. In view of the small sample size, these findings cannot be considered statistically robust, but the authors suggest that further research about the effect of these cells on the GVL effect are warranted, as are studies designed to define the optimal provenance of MSCs (same donor as HSCs or third party). Finally, co-transplantation of MSCs and HSCs may be a “double-edged sword”. As Table 2 shows, some studies reported unsatisfactory outcomes [53, 55, 56], but further randomized clinical trials are required to assess the risk of blunting the GVL effect when MSCs are co-transplanted with HSCs, particularly to determine the optimal timing of MSC infusion for aGVHD prophylaxis—days after HSC infusion or at the engraftment, without affecting GVL.

**Table 2:** Clinical studies of MSCs for treatment of acute graft-versus-host disease

References	Study type	*N*	MSC source	P/Media	MSC dose/kg	Response
Lazarus et al. [53]	Safety and feasibility	46	BM; related donor	–/FBS	1.0–5.0 × 109 (6) 4 h before HSCT	28 % (13) developed grade II–IV aGVHD. 61 % (36) developed cGVHD
Le Blanc et al. [55]	Pilot	7	BM; HLA-identical and HLA-haploidentical	2–3/FBS	1.0 × 10 (6)	5 patients developed aGVHD and 1 developed cGVHD.
Ball et al. [126]	Pilot	47	BM; HLA-matched	3/FBS	1.0–5.0 × 10 (6) 4 h before HSCT	4 deaths (2 relapses, 2 viral infections)
Ning et al. [54]	Phase II, randomized	10	PBSC or BM; related donors	–/FBS	0.03 × 10 (6) a 1.5 × 10 (6)	The incidence of grade II–IV GVHD was 11.1 % in the MSC group versus 53.3 % in the control group. The incidence of aGVHD was 44.4 % in the MSC group versus 73.3 % in the control group
Guo et al. [127]	Phase II (???)	33	PBSCT + MSCs	3–5/FBS	0.5 × 10 (5) a 1.7 × 10 (6)	15 patients (45.5 %) developed grade I-IV aGVHD and 2 (6.1 %) developed grade III–IV aGVHD. 9 (31 %) of 29 developed cGVHD
Zhang et al. [128]	Safety and feasibility	14	BM; HLA-identical (same donor as HSCT)	3–4/human MSC stimulatory supplements	2.0 × 10(6) após 1 hora do TCTH	Two patients developed grade I–IV aGVHD and two developed cGVHD. Four patients contracted CMV infection and were treated successfully. Five patients died
Gonzalo-Daganzo et al. [129]	Pilot	9	BM; third party	1–3/FBS	1.04–2.15 × 10 (6) immediately after cord blood and mobilized HSC transplantation	Four patients developed grade II aGVHD (two had steroid-refractory GVHD).
Macmillan et al. [130]	Phase I-II	15	BM; related donors	1–4/–	0.9–5 × 10 (6) on day of HSCT; 3 patients received a booster dose 21 days later	Incidence of GVHD at 100 days post-HSCT was similar in patients who received MSCs (*p* = 0.44)
Fang et al. [131]	Report of two cases	2	Adipose tissue	–/FBS	1.0 × 10 (6) within 4 h after PBSC infusion	Both patients survived 2 years post-transplantation.
Baron et al. [34]	Pilot	20	PBSC of HLA-mismatched donors	P2/FBS	1.0–2.0 × 10 (6), 30–120 min before PBSCT	Co-transplantation of HLA-mismatched PBSCs and third-party MSCs was feasible. No mortality (10 % at 1-year follow-up)
Bernardo et al. [56]	Cohort	13	BM; related donor	2–3/FBS	1 a 3.9 × 10 (6) on the day of HSCT	No differences between the intervention and control groups

## Mode of action of MSCs as cell therapy agents

MSCs are known to interact with other immune system cells; however, the mechanisms underlying their immunomodulatory action have yet to be fully elucidated. MSCs are capable of interacting primarily with natural killer (NK) cells, monocytes, and regulatory T cells [57]. These cells also inhibit the immune response by means of complex mechanisms, including changes in antigen-presenting cell maturation and suppression of monocyte-derived dendritic cell (DC) differentiation and activity. Furthermore, MSCs alter the cytokine secretion profiles of effector T cells, DCs, and NK cells, shifting it from a pro-inflammatory Th1 cytokine profile to an anti-inflammatory Th2 cytokine profile. These effects may prove useful in the prevention and treatment of GVHD and in the inhibition of graft rejection [58]. However, it bears stressing that these findings are mostly derived from in vitro studies, as there has been little in vivo research.

The clinical use of MSCs requires an understanding of the biological characteristics that underlie their therapeutic effects. Four properties or MSCs are currently considered most important to potential clinical uses: (1) their ability to migrate to sites of inflammation when injected intravenously; (2) the ability to differentiate into various cell types; (3) the ability to secrete multiple bioactive molecules capable of inhibiting inflammation and healing injured cells and (4) the ability to perform immunomodulatory functions while lacking immunogenicity [59].

## Homing and engraftment of MSCs

In vitro and animal model studies have showed that culture-expanded MSCs are capable of homing to and grafting into sites of inflammation and exerting functional effects on local tissues after systemic administration. Cell migration depends on a variety of stimulatory or regulatory signals, which range from growth factors to chemokines secreted by damaged cells and/or respondent immune cells [60]. Studies have shown that MSC homing is controlled by a wide range of growth factor receptor tyrosine kinases, such as platelet-derived growth factor (PDGF) or insulin-like growth factor-1 (IGF-1), and chemokines such as CCR2, CCR3, CCR4, CCR7 and CCL5, as shown by in vitro homing assays [18, 61].

## Secretion of bioactive molecules by MSCs

MSCs can also secrete a wide range of bioactive molecules, including growth factors, cytokines, and chemokines, which can exert dynamic effects on specific sites. Table 3 lists several of these molecules and their respective roles. In a protein array study of MSCs, Parekkadan et al. [62] detected 69 of 174 analyzed proteins. Most of the detected molecules were growth factors, cytokines, and chemokines, which are known to have regenerative and anti-apoptotic effects.

**Table 3:** Molecules secreted by MSCs and their roles

Molecule	Role
Prostaglandin E2 (PGE2)	Mediates antiproliferative [132], anti-inflammatory [133] effects
Interleukin 10 (IL-10)	Anti-inflammatory [36]
Transforming growth factor beta 1 (TGFβ1), hepatocyte growth factor (HGF)	Suppress T-cell proliferation [80]
Interleukin 1 receptor antagonist	Anti-inflammatory [134]
Human leukocyte antigen, G isoform (HLA-G5)	Suppresses naive T-cell proliferation [118]
Antimicrobial peptide LL-37	Antimicrobial, anti-inflammatory [135]
Angiopoietin 1	Restores epithelial protein permeability [136]
Matrix metalloproteinases 3 and 9 (MMP3, MMP9)	Mediate neovascularization [137]
Keratinocyte growth factor	Alveolar epithelial fluid transport [138]
Vascular endothelial growth factor (VEGF), basic fibroblast growth factor (bFGF), placental growth factor (PlGF), monocyte chemotactic protein 1 (MCP-1)	Increase endothelial cell and smooth muscle cell proliferation [139], [140]

## Immunomodulatory effects of MSCs

The ability of MSCs to modulate immune function was first recognized in 2000, when Liechty et al. found that MSCs have immunomodulatory properties that enabled the persistence of human MSCs in a xenogeneic environment [63, 64]. Several later studies gradually confirmed these immunomodulatory properties. Nevertheless, the precise mechanisms underlying MSC-mediated immunomodulation have yet to be fully understood. Cell–cell contact and release of soluble immunosuppressant factors are the main mechanisms being studied since, as aforementioned, MSCs are capable of interacting with a wide range of immune system cells, including T lymphocytes, B lymphocytes, natural killer cells, dendritic cells, and macrophages. Table 4 lists the main immunomodulatory effects of MSCs on these immune cells.

**Table 4:** Immunomodulatory effects of MSC therapy on immune system cells

Cell type	Effects of MSC therapy	Soluble factors involved
T lymphocytes	Suppresses T-cell proliferation induced by cellular or nonspecific mitogenic stimuli [80]; Alters the cytokine secretion profile of effector T cells [77]; Promotes expansion and activity of regulatory T cells [87] Induces apoptosis of activated T cells [141] Regulatory T-cell generation [89]	IL-1β [90] TGFβ1 [90] HGF [80] PGE2 [77] IDO [141] LIF [93] IGF [94] HLAG [93] CCL1 [89]
B lymphocytes	Inhibits B cell proliferation [103]; Affects the chemotactic properties of B cells [101]; Suppresses B cell differentiation [142]	IFN-γ [102] IL-6 [99]
NK cells	Alters the NK cell phenotype, suppresses NK cell proliferation, cytokine secretion, and cytotoxicity against HLA class I-expressing targets [115]	TGFβ [115] IDO [116] HLAG5 [118] PGE2 [116]
Dendritic cells (DC)	Influences the differentiation, maturation, and role of DCs differentiated from monocytes [143]; Suppresses DC migration, maturation, and antigen presentation [144]	M-CSF [105]
Macrophages	M2 macrophage recruitment; Conversion of pro-inflammatory M1 macrophages into anti-inflammatory M2 macrophages; Attenuates macrophage inflammatory response	CCL3 [119] CCL12 [119] CXCL2 [119] PGE2 [119] KYN [119] TSG6 [119]

MSCs appear to exhibit little immunogenicity, in view of their low class I MHC expression and absence of class II MHC molecules. Furthermore, MSCs do not express co-stimulatory molecules such as CD40, CD80, or CD86, which are involved in T-cell activation in the transplant rejection setting [65, 66]. Several studies have shown that differentiated and undifferentiated MSCs have alloantigen suppressing effects on in vitro myogen-induced lymphocyte proliferation using mixed lymphocyte reaction (MLR) cultures, with a concomitant decrease in secretion of pro-inflammatory cytokines such as interferon gamma (IFN-γ) and tumor necrosis factor-alpha (TNF-α) [65, 67, 68].

Human MSCs have been reported to express toll-like receptor (TLR) types TLR1 to TLR1 [69–73]. These receptors are associated with tissue injury and infection. Furthermore, baseline levels of TLR expression in BM-derived and adipose tissue-derived human MSCs are sensitive to environmental stimuli. Expression may be hyper-regulated by hypoxia (for TLR1, TLR2, TLR5, and TLR9) or inflammatory conditions, by IFNγ, TNF, IFNα, and IL-1β (for TLR2, TLR3, and TLR4) [74, 75].

In a non-inflammatory environment, MSCs express low levels of cyclooxygenase 2 (COX-2), prostaglandin E2 (PGE2), transforming growth factor-β (TGF-β), indoleamines (IDO), and other factors. However, pro-inflammatory cytokines drastically regulate secretion of anti-inflammatory factors by MSCs [76]. One example is increased secretion of IDO, hepatocyte growth factor (HGF), and TGF-β induced by IFN-γ and increased secretion of PGE2 induced by TNF-α in MSCs [77–79].

## Interactions between MSCs and T lymphocytes

The interactions between MSCs and T lymphocytes have been most widely studied, particularly in vitro. Several articles have reported that MSCs have an impact on several T-cell properties—for instance, suppressing proliferation of activated CD4^+^ (helper) T cells and CD8^+^ (cytotoxic) T cells [80, 81]. MSCs keep activated T cells in phase G0/G1 of the cell cycle [82], but apoptosis is not induced [80, 81]. In addition to their ability to regulate activated T-cell proliferation, MSCs may prolong survival of unstimulated T cells and inhibit endogenous proteases involved in cell death [83]. Other studies have shown that MSCs reduce expression of IFN-γ by CD4^+^ Th1 cells and IL-12 release by CD4^+^ Th17 (T helper) cells, whereas IL-4 secretion by CD4^+^ Th2 cells is increased [77, 84]. The cytolytic potential of cytotoxic T cells may also be affected by MSCs [85].

Recent studies have investigated the impact of MSCs on regulatory T lymphocytes (T_reg_), a population of CD4^+^/CD25^high^ cells that play an important role in induction of peripheral tolerance and inhibition of pro-inflammatory immune responses [86]. Many studies have shown that MSC can induce expansion of CD4^+^/CD25^high^/Foxp3^+^ T cells (functional T_regs_) [77, 84, 87, 88]. Countless mechanisms have been suggested, including cell contact-dependent and independent mechanisms, but there is no consensus; TGF-β, for instance, was reported to be involved in this effect by English et al. [87], but not in a later study conducted by Prevosto et al. [88]. This discrepancy is probably attributable to phenotypic variation and differences in MSC culture methods.

The current body of evidence provides an outline of which specific molecules are involved in the immunomodulatory effects of MSCs on effector T lymphocyte proliferation and function. In the human immune system, the effects of MSCs on T cells are mediated primarily by independent cell–cell contact, evincing the importance of secretion of such factors as [89] IL-1β [90], TGFβ-1 [87], HGF [80], PGE2[77], IDO[91], heme oxygenase-1 (HO-1)[92], leukemia inhibitory factor (LIF) [93], IGF [94], sHLA-G5 [93], galectin [95], and Jagged-1 [69].

Some authors have reported that, in vitro, pretreatment of MSCs with the pro-inflammatory cytokine IFN-γ boosts immunomodulation [96, 97]. This may explain the ability of MSCs to act on inflammatory conditions such as aGVHD, in which production of cytokines such as IFN-γ by T lymphocytes and NK cells may promote MSC immunomodulation, and, subsequently, suppress CD4^+^, CD8^+^, and NK cell proliferation [97]. This paradox must still be elucidated if the immunomodulatory effects of MSCs are to be understood [96, 98].

## Interactions between MSCs and B lymphocytes

Some studies have shown that MSCs can regulate B lymphocyte functions, including migration, proliferation, and immunoglobulin (Ig) synthesis [99]. In vitro, MSCs inhibit B lymphocyte proliferation by G0/G1 cell cycle arrest. MSCs also inhibit IgM, IgA, and IgG production [100, 101]. The effects of MSCs on B lymphocytes are mediated both by soluble factors, such as IFNγ and IL-6 [99, 102], and by cell–cell (MSC–B lymphocyte) contact [99, 103].

## Interactions between MSCs and DCs

DCs are derived from monocytes and lymphoid precursors, and function as potent antigen-presenting cells (APCs), which internalize, transport, and present antigens to naïve T cells, thus triggering T lymphocyte activation [104]. In the presence of MSCs, differentiation of CD14^+^ monocytes into DCs is impaired, and monocytes retain high CD14^+^ expression—a marker of DC immaturity—without an increase in CD1a, HLA-DR, or co-stimulatory molecule expression, thus preventing DCs from inducing effector T-cell response [105]. MSCs also suppress the T-cell-activating effect of DC, including stimulation of T lymphocyte proliferation, reduction of Th1 differentiation from naive CD4 + T cells, and promotion of Th2 responses. MSCs may reduce secretion of TNF-α by DCs, which leads to a quantitative decrease in the production of IFNγ-expressing Th1 cells. APCs generated in the presence of MSCs express low levels of IL-12, TNF-α, and MHC II and high concentrations of IL-1β and IL-10, independently of CD86 expression [106]. MSCs also induce DCs to secrete IL-10, which skews the immune response profile toward T_regs_ and IL-4-producing Th2 cells [77]. Furthermore, MSCs hinder cytokine release by activated DCs through PGE2 [77, 107].

Some studies have shown that MSCs may function as non-professional APCs, as IFNγ-stimulated MSCs have been reported to present exogenous antigens by means of MHC II overexpression, triggering CD4^+^ T-cell activation [108, 109]. MSCs can also induce CD8^+^ T-cell proliferation by presenting endogenous antigens [110, 111]. Studies have also shown that, like DCs, MSCs express high levels of TLR. TLRs are primary receptors expressed by APCs that recognize pathogen-associated molecular patterns. Triggering of TLR3, which binds double-stranded RNA, and TLR4, which binds lipopolysaccharides (LPS) and innate autoantigens, leads to production of pro-inflammatory mediators such as IL-1b, IL-6, and IL-8 [69, 112]. However, another study has shown that TLR triggering by MSCs induces immunosuppression, thus leading to production of immunosuppressant kynurenine induced by the IDO enzyme [71]. These conflicting data, which suggest that MSCs both suppress DC maturation and are themselves APCs and thus induce a pro-inflammatory response, justify further research.

## Interactions between MSCs and NK cells

NK cells are lymphocytes of the innate immune system and play an important role in the elimination of virally infected and tumor cells. When activated, NK cells are highly cytotoxic and secrete large quantities of pro-inflammatory cytokines, such as TNF-α and IFN-γ [113, 114]. Within the innate immune response, these cytotoxic lymphocytes recognize and respond to MHC molecules, distinguishing self from non-self by means of co-stimulatory and inhibitory receptors instead of specific antigens, as done by adaptive immune system T and B cells. A small number of studies have shown that MSCs are able to suppress NK cell proliferation and cytokine production [77, 85, 115]. This inhibition requires both cell–cell contact and secretion of soluble factors, such as PGE2 and TGF-β [97, 115]. MSCs are also capable of modulating NK cell cytotoxicity by decreasing the amount of cytokines (including IFN-γ, IL-10, and TNF-α) secreted by NK cells; this phenomenon also requires cell–cell contact [115, 116]. Nevertheless, stimulated NK cells can still lyse both autologous and allogeneic MSCs [115, 117]. The NK cell-activating receptors NKp30, NKG2D, and DNAM-1 act as mediators of NK cell-versus-MSC cytotoxicity. Conversely, IFN-γ stimulates MSCs, thus decreasing their susceptibility to lysis by NK cells by increasing surface expression of MHC I [117]. Finally, secretion of soluble HLA-G (sHLA-G) by MSCs plays an important role in inhibiting NK cell cytotoxicity and IFN-γ release [118].

## Interactions between MSCs and macrophages

Macrophages play a crucial role in the inflammatory response and in tissue regeneration. Macrophages differentiate from monocytes after migration into tissues under homeostatic conditions or after inflammatory activation [119]. In a CCL3- and CXCL2 (MIP2)-dependent manner, human MSCs recruit monocytes and macrophages from across the body and into inflamed tissues [120]. Macrophages exhibit plasticity, and can be polarized by the microenvironment (including by presence of MSCs) into two forms of activated macrophages, M1 (antimicrobial) or M2 (inflammatory). Co-culture of human MSCs and monocytes promotes formation of M2 macrophages, which exhibit high levels of IL-10 expression and intense phagocytic activity and low TNF and IFNγ levels and MHCII expression [121–123]. This differentiation occurs as a result of cell–cell contact and by several soluble factor-mediated mechanisms, such as IDO and PGE2 secretion by MSCs.

## Conclusion

In recent years, MSCs have become a subject of clinical research interest due to their easy isolation and culture, striking potential for differentiation, production of growth factors and cytokines, and potential immunomodulatory effects. Upon direct contact with tissues or by means of paracrine interactions, MSCs trigger the release of a variety of soluble factors that act on immune system cells. These cells are able to identify “warning signs”, or a lack thereof, and modulate immune cells accordingly so as to ensure a balance between activation and inhibition of immune responses as necessary. Nevertheless, the mechanism whereby MSCs exert immunosuppressant effects on the inflammatory response and on transplant rejection processes has yet to be fully elucidated in vivo, despite extensive in vitro research.

In short, MSCs appear to be safe and well-tolerated for use in cell therapy, and, as a treatment modality, can provide hope to patients with steroid-resistant aGVHD. The results of clinical trials have thus far been encouraging, and research in this field continues to improve. At the time of writing, 307 clinical trials of mesenchymal stem cells for a variety of therapeutic applications were registered in the ClinicalTrials.gov database (http://clinicaltrials.gov), 31 of which designed to test MSCs in the treatment of GVHD. Further information on the biology of MSCs must be obtained so as to keep pace with progress being made in regenerative medicine and ascertain the safety and efficacy of these cells for treatment of aGVHD and other inflammatory and autoimmune conditions.
